# Adjusting risk-taking to the annual cycle of long-distance migratory birds

**DOI:** 10.1038/s41598-018-32252-1

**Published:** 2018-09-18

**Authors:** Peter Mikula, Mario Díaz, Tomáš Albrecht, Jukka Jokimäki, Marja-Liisa Kaisanlahti-Jokimäki, Gal Kroitero, Anders Pape Møller, Piotr Tryjanowski, Reuven Yosef, Martin Hromada

**Affiliations:** 1 Department of Zoology, Faculty of Science, Charles University, Viničná 7, 128 43 Praha 2, Czech Republic; 2 Department of Biogeography and Global Change, Museo Nacional de Ciencias Naturales, CSIC, c/Serrano 115bis, E-28006 Madrid, Spain; 3 Institute of Vertebrate Biology, Czech Academy of Sciences, Květná 8, 603 65 Brno, Czech Republic; 4 Arctic Centre, University of Lapland, PO Box 122, 96101 Rovaniemi, Finland; 5 Rabin High School, Yotam Street 51, Eilat, 8820301 Israel; 6 Ecologie Systématique Evolution, Université Paris-Sud, CNRS, AgroParisTech, Université Paris-Saclay, F-91405 Orsay Cedex, France; 7 Institute of Zoology, Poznań University of Life Sciences, Wojska Polskiego 71C, 60-625 Poznań, Poland; 8 Ben Gurion University of the Negev – Eilat Campus, P. O. Box 272, Eilat, 88000 Israel; 9 Laboratory and Museum of Evolutionary Ecology, Department of Ecology, Faculty of Humanities and Natural Sciences, University of Presov, 17 novembra 1, 080 01 Prešov, Slovakia; 10 Faculty of Biological Sciences, University of Zielona Góra, Prof. Z. Szafrana 1, 65-516 Zielona Góra, Poland

## Abstract

Life-history theory predicts that current behaviour affects future reproduction, implying that animals should optimise their escape strategies to reflect fitness costs and benefits of premature escape. Both costs and benefits of escape may change temporally with important consequences for the evolution of escape strategies. Moreover, escape strategies of species may differ according to their positions on slow–fast pace of life gradients. We studied risk-taking in long-distance migratory animals, waders (Charadriiformes), during the annual cycle, i.e., breeding in Europe, stopover in the Middle East and wintering in tropical Africa. Phylogenetically informed comparative analyses revealed that risk-taking (measured as flight initiation distance, FID) changed significantly over the year, being lowest during breeding and peaking at stopover sites. Similarly, relationships between risk-taking and life-history traits changed among stages of the annual cycle. While risk-taking significantly decreased with increasing body mass during breeding, risk-taking–body mass relationship became marginally significant in winter and disappeared during migration. The positive trend of risk-taking along slow–fast pace of life gradient measured as adult survival was only found during breeding. The season-dependent relationships between risk-taking and life history traits suggest that migrating animals respond to fluctuating environments by adopting behavioural plasticity.

## Introduction

Behaviour is considered to be the most important mechanism by which animals flexibly cope with fluctuating environmental patterns and processes^1–5^. Among environmental factors, predation is a major cause of extrinsic mortality in animals, and it is considered an important selective force in the evolution of adaptive traits in all organisms, including birds^1,6,7^. Optimal escape theory predicts that animals under the risk of predation optimise their escape strategy and make a trade-off between the fitness-related benefits from staying and the costs of fleeing^4,5,8,9^. The more tolerant animals increase their mortality risk, resulting in reduced future reproductive success, whereas increased vigilance results in increased monitoring, metabolic costs and reduced food intake^10,11^. However, both costs and benefits of escape might vary temporally and spatially, with important consequences for the evolution of temporally variable antipredator strategies^7,12–14^. The season-dependent aspect of decision-making has been largely ignored with only a few empirical studies of wild living animals^15^.

During breeding, birds may be more susceptible to taking risks, as adults are forced to collect sufficient food for their young. Moreover, increased risk-taking may be linked to increased secretion of steroid hormones that is associated with increased inter- and intraspecific aggression in both sexes^16,17^ or aspects of nest defence, such as the ‘broken wing’ behaviour attracting potential predators^18^. Alternatively, birds may be more cautious as they need to finish breeding by minimising the probability that their nests will be found by predators^7^. This may also be accompanied by physiological changes such as an increased level of glucocorticoids, usually resulting in an increase in vigilance activities that are likely to affect escape behaviour in birds^19^. Similarly, during the winter season, birds may be risk-averse as there are no clear advantages to excessive risk-taking.

Seasonally climatic changes affect birds through a variety of processes, including, for instance, predation, resource availability, and competition during the year, and many animals cope with this environmental seasonality by migration^20^. Migratory birds have high metabolic rates due to their energetically expensive way of life and often expend significant amounts of their energy deposits during migration^21,22^. Birds on migration are typically forced to make short-term stopovers to rest, drink or feed in order to accumulate energy deposits to successfully complete migration^23,24^. Variation in an animal’s energetic state and energy stores affects individual willingness to pay the costs of antipredator responses, especially with animals in worse body conditions showing weaker antipredator responses^13,14,25^. Hence, it is expected that birds at stopover sites should maximise risk-taking by reducing their escape distance to a minimum. However, need for efficient foraging while simultaneously keeping monitoring costs for the presence of predators at acceptable rates could be accompanied by the development of other antipredator strategies such as flocking^26,27^.

Flocking has traditionally been recognised as an effective antipredator strategy, with individuals in larger flocks having a reduced probability of being killed^26–29^. Birds in large flocks minimise monitoring costs per capita by increased collective vigilance but flocking may also improve the probability of finding new foraging patches and simultaneously to tolerate higher levels of disturbance^10,30,31^. Hence, birds are expected to form large flocks during periods when facing increased predation risk, decreased food availability and/or increased energy demands. Flock size is also predicted to be an important determinant of individual vigilance when approached by a predator. Firstly, birds in larger flocks may be associated with longer escape distances as their escape response may depend on the response of the most risk-sensitive individual in the flock, and several individuals will detect danger earlier than a single individual^4,28^. In contrast, individual vigilance may be negatively correlated with flock size due to the ‘dilution’ or ‘many eyes’ effects^30,32–34^. While the flight-prone strategy would prioritise reduction of risk, the flight-delaying strategy permits birds to maximise the benefits of delayed escape.

According to their life-history traits, animals can be aligned along a slow–fast pace of life axis, some prioritizing high survival (slow pace of life) while others prioritize reproduction (fast pace of life)^35–37^. This reflects a trade-off between survival and reproductive potential of individuals because it is impossible to simultaneously maximize gains from both strategies^38^. For instance, large bodied animals typically live longer, and invest comparatively more into survival and self-maintenance, but reduce their investment into current reproduction^39,40^. Life-history theory predicts that current behaviour affects future actions, implying that species having a higher probability of adult survival should tolerate lower risk because the prospect of future reproduction is high^8,9,36,38,41^. However, the costs and benefits of escape during the season may change dynamically, presumably having an influence on relationships between escape strategy and life-history traits across the year. Despite growing interest in the importance of the slow–fast pace of life for understanding the interactions between animals and the environment^37,41^, empirical evidence for the relationship between behavioural traits, such as escape behaviour, and life-history in animals under fluctuating environmental conditions is still scarce.

Here, we focused on temporal changes in risk-taking in a model group of animals, waders (Charadriiformes), an ecologically diverse group of species having diverse life-histories, including body size, investments into current reproduction and survival rate^42,43^. Waders are also textbook long-distance migrants, typically breeding in temperate to polar climatic zones but undergoing spectacular annual migrations to wintering grounds in tropical or subtropical regions^42^. We studied risk-taking during the most prominent parts of the annual cycle of waders, i.e. breeding in Europe, stopover in the Middle East during the spring migration and wintering in tropical Africa. The level of risk-taking was estimated by measuring the ‘flight initiation distance’ (FID), the distance at which individuals escape when approached under standardised conditions^4,5,9,30,36^. We examined whether (1) risk-taking varies during the annual cycle due to temporal changes in the costs and benefits of premature escape; (2) flock size change dynamically over the season, and (3) species with different position of slow–fast pace of life axis differs in terms of risk-taking and whether the relationship between risk-taking and life-history varies temporally.

## Results

### Summary statistics

FID data were collected for 10,877 individuals from 20 wader species, recorded during at least two stages of their annual migration cycle. Data for all three stages (i.e. breeding, migration and wintering) were available for eight species. Only single FID estimates for birds within the same flock were used to avoid problems of statistical dependence, thus reducing the total number of estimates to 2,072 FIDs from 115 populations (Appendix 1). The number of measurements per species ranged from 2 to 619 (mean ± standard deviation: 104 ± 181, median = 30). We obtained 287 measurements (20 species) from breeding areas (Europe), 1443 (10 species) from the spring migration stopover site (Israel), and 342 (18 species) from wintering areas (tropical Africa). Log-transformed FIDs were highly repeatable within species (intra-class correlation r = 0.997; F_19,2053_ = 18.65, P < 0.001).

### FID and SD, flock size and human activity

FID was significantly positively correlated with SD and negatively with flock size, but not to human activity (supplementary Table S1). Effect sizes were large for SD (r = 0.609) and moderate for flock size (r = 0.349). Mean flock size changed among stages of the annual cycle (B = −0.26, SE (B) = 0.05, t = −5.52, P < 0.001), with no significant phylogenetic (λ = 0.141; χ^2^_112_ = 0.03. P = 0.869) or sample size (V + 1*W) effects. Mean flock sizes were significantly larger during migration (mean ± SE = 0.46 ± 0.10) than during winter (mean ± SE = 0.04 ± 0.05) (Tukey test, P < 0.001), during winter than during breeding (mean ± SE = −0.12 ± 0.02) (Tukey test, P = 0.018), and during migration than during breeding (Tukey test, P < 0.001).

### FID and season and species traits

After correcting for SD and flock size effects, mean FID varied significantly between life cycle stages, with moderate effect sizes (Table 1). Corrected FIDs (residuals from models including confounding variables and phylogenetic and sample size effects) were shorter at stopover sites (mean ± SE = −0.06 ± 0.02) than during breeding (mean ± SE = 0.05 ± 0.02) (Tukey test, P = 0.003). Stopover FIDs did not differ from winter FIDs (mean ± SE = −0.04 ± 0.02) (Tukey test, P = 0.946) and winter FIDs were marginally shorter than breeding FIDs (Tukey test, P = 0.073).

**Table 1:** Results of a phylogenetic generalized least square regression (PGLS) model testing for among-stage differences in flight initiation distance (FID) after accounting for the confounding effects of starting distance (SD) and flock size as well as for phylogenetic (λ = 0.476; χ^2^_112_ = 12.89; P = <0.001) and sample size (V + 20 W) effects.

Effect	B	SE(B)	t	P	r
SD	0.42	0.05	8.64	**<0.001**	0.537
Flock size	−0.02	0.04	−0.40	0.68941	0.038
Stage	0.09	0.02	4.44	**<0.001**	0.391

Model statistics are F_4,112_ = 31.77, P < 0.001, R^2^ = 0.45, AIC = −132.4. Boldface indicates statistical significance, and Pearson r values effect sizes.

**Figure 1:**
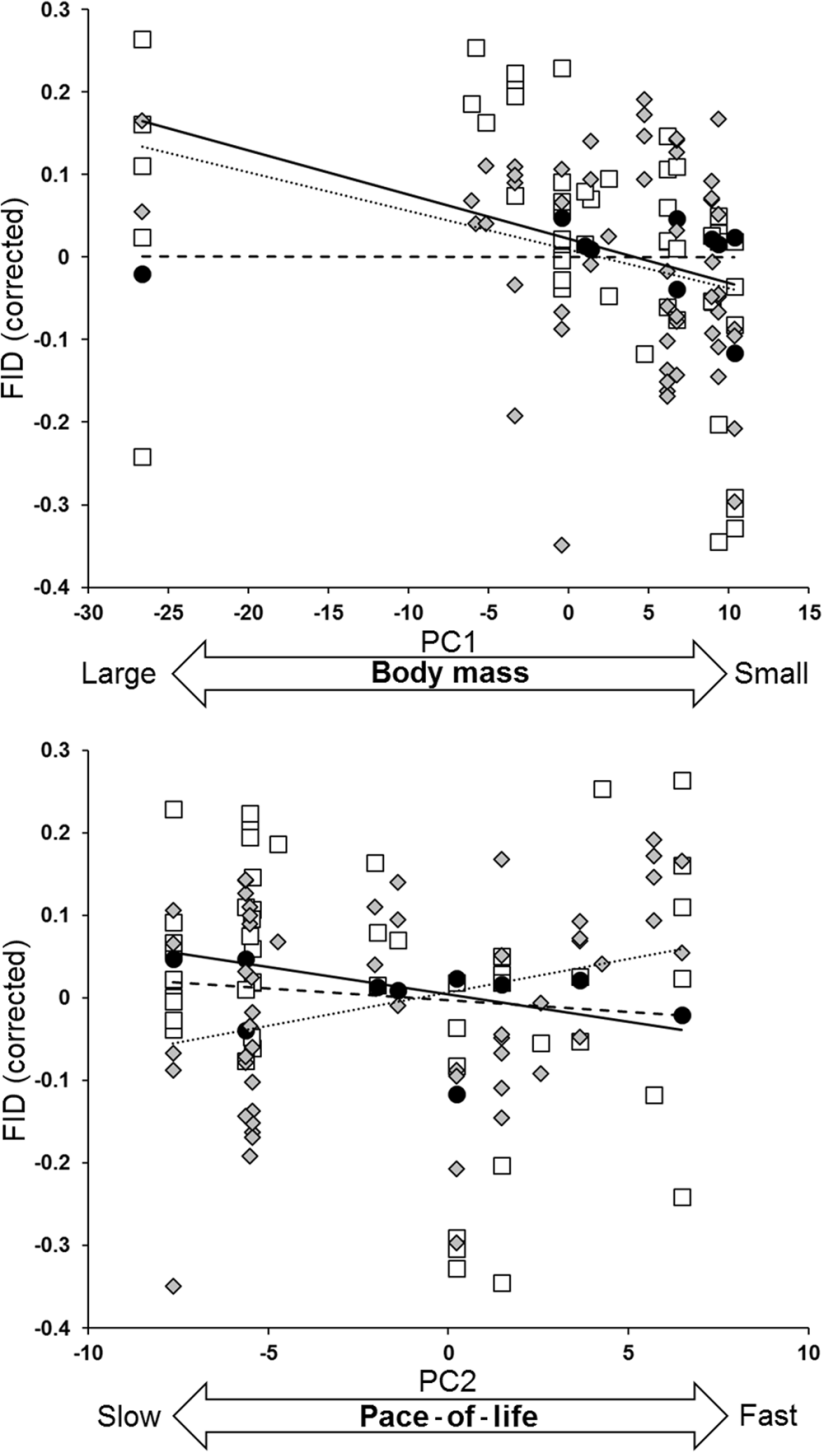
Changing relationships between flight initiation distance (FID) and body mass (PC1) and slow–fast pace of life (PC2) gradients between stages of the annual cycle (breeding = open squares, continuous line; migration = filled dots, dashed line; winter = grey diamonds, dotted line). Lines are linear regression lines computed by means of phylogenetically corrected regressions accounting for phylogeny, sample size, starting distance and flock size effects. B (±SE) and P values are: PC1, breeding: −0.009 (±0.002), P < 0.001; PC1, migration: −0.001 (±0.002), P = 0.498; PC1, winter: −0.006 (±0.003), P = 0.056; PC2, breeding: −0.011 (±0.004), P = 0.008; PC2, migration: −0.008 (±0.005), P = 0.150; PC2, winter: 0.007 (±0.004), P = 0.097.

FID–life history relationships changed among stages of the annual cycle (Table 2, Fig. 1). Significant negative FID–PC1 relationships during breeding (meaning positive FID–body mass relationships as PC1 was an inverse gradient of body mass) became marginally significant in winter and disappeared during migration. Negative trend of FID along slow–fast pace of life gradients (PC2) was only found during breeding. This trend disappeared during winter and migration.

**Table 2:** Results of a phylogenetic generalized least square regression (PGLS) model testing for relationships between flight initiation distance (FID), life-history traits (summarized in two principal components corrected for phylogeny, PC1 and PC2) and its variation among stages of the annual cycle, after accounting for the potential effects of starting distance (SD) and flock size as well as for phylogenetic (λ = 0.320; χ^2^_115_ = 3.57, P = 0.059) and sample size (V + 100 W) effects.

Effect	B	SE (B)	t	P	r
SD	0.401	0.047	8.603	**<0.001**	0.626
Flock size	−0.014	0.036	−0.391	0.697	0.037
PC1	−0.001	0.003	−0.532	0.596	0.050
PC2	0.006	0.006	1.049	0.296	0.098
Stage	0.095	0.019	5.085	**<0.001**	0.431
PC1 x stage	−0.004	0.002	−2.162	**0.033**	0.199
PC2 x stage	−0.005	0.004	−1.282	0.202	0.120

Model statistics are F_8,115_ = 21.82, P < 0.001, R^2^ = 0.56, AIC = −145.99. PC1 was an inverse gradient of body mass, and PC2 can be interpreted as a fast–slow pace of life gradient. Boldface indicates statistical significance, and Pearson r values effect sizes.

## Discussion

Risk-taking measured as FID changed significantly over the annual cycle, being lowest during breeding and highest at stopover site and during winter, probably reflecting responses to environmental variation. Recent life-history theory has attempted to understand how organisms adjust their behaviour to maximise fitness in fluctuating environments. Our results revealed that relationships between risk-taking and life-history traits changed among stages of the annual cycle, being relevant at some stages but not at others. As in numerous other animals, birds adjusted their escape strategies to a range of intrinsic (e.g. body mass) and extrinsic factors (e.g. risk of predation). Though rarely empirically studied in wild-living migratory animals, we support the idea that risk-taking by animals is temporally variable and adjusted to annual changes in the relative costs and benefits of such behaviour.

Migratory animals face different challenges during their annual migration cycle, including changes in the predator community along the migration route. In general, predator diversity is low at high latitudes but increases towards the tropics^40,44,45^. On a large scale, birds typically respond to the increased abundance of predators by decreased risk-taking^5^. We found that birds were risk-averse mainly during breeding in temperate zones, probably so that they minimised the probability of their death due to predation and their nest being exposed to predators^7^. However, waders took relatively high risks when wintering in tropical Africa, where predator communities are expected to be more diverse and abundant than in the temperate zone^5,36,45^, and when no clear advantage of excessive risk-taking is assumed. Interestingly, risk-taking was the greatest on stop-over sites where birds can face extensive mortality due to predation from migratory or resident raptors and/or disturbance by humans^46–49^. This pattern may be explained by energetic state and amount of available energy stores of individuals^14,25^. Birds on migration suffer from extensive loss of fat stores and organ shrinkage and are in worse physical condition than during the sedentary periods of the year^50^. Animals suffering food deprivation display considerably lower levels of antipredator response than animals in favourable conditions and this effect may be amplified when suitable foraging stopover sites are rare^14,25^. A high food intake at stop-over sites is crucial for the success of long-distance migration and migratory birds may be forced to take greater risks. Although, alternatively, birds may prolong their stay at the stopover site to collect sufficient amounts of food, it seems that waders make a rather short rest in Eilat^24^, possibly because of high numbers of concurrent migrating raptors^48,49,51^. This also suggests that the costs of insufficient food intake at stop-over sites are higher than those introduced by delayed antipredator response. The patterns found are also consistent with physiological changes occurring during the annual cycle in birds. For instance, levels of glucocorticoids, that are positively linked to vigilance in birds, is typically elevated during breeding but falls towards post- and pre-breeding parts of the year^19^. Other explanation is that arriving birds are already exhausted after a long journey, and still in the process of re-assimilating their digestive tract, and thus escape only when absolutely necessary^52,53^. In summary, annual patterns of FID can be due to variation in physiology over the annual life cycle, while some variation could also be due to current environmental conditions. We tried to measure FIDs under ‘standardized’ environmental conditions at the stopover site, even controlling for the potential effect of hunting pressure by selecting a site where legal and illegal hunting is not allowed/rarely occur^54^, as in breeding and wintering study sites (pers. obs.). This restriction, as well as logistical constraints, explains why we only recorded data for one stopover site during only one study year. This fact may have biased the results if short-term environmental conditions were the main determinants of FIDs. Despite that migratory birds exhibit high inter-annual consistency in FID, even after extreme weather conditions^55^, we still could not be sure that this is also the case during the migration period because our stopover data were only collected during one year. Hence, this limitation must be taken into account when assessing our study. Further research is clearly necessary for analysing short- and long-term (across years) changes in FID during migration in general, and at stop-over sites in particular.

Flock size in waders varied significantly between stages of the annual cycle, being largest at stopover sites, suggesting that waders cope with increased predation risk by grouping behaviour^29^. Group size is often correlated with risk-taking but with no consistent among taxonomic group effect^4^; in waders, it seems that flock size has a negative effect on risk-taking^4,28^. However, when tested separately, there was a significant positive relationship between risk-taking and flock (see supplementary Table S1), suggesting that the ‘dilution’ effect or the ‘many eyes’ effect allow waders in our dataset to maximize their benefits of staying put^30,32,33^. However, in the full model, we failed to find any overall effect of flock size on risk-taking. This is probably linked to flocking behaviour during the year in waders. While they typically live in pairs or solitarily when breeding, especially at stopover sites, birds move in large flocks, probably irrespective of their escape distance. Thus, it is likely that despite mortality due to predation remains high at stopover sites^56^, flocking may have evolved as an alternative antipredator strategy, preventing excessive mortality at sites with high predation risk while keeping foraging time to an acceptable level. This minimisation of monitoring costs per capita by increased collective vigilance in larger flocks is consistent with the framework proposed by the FEAR (Flush Early and Avoid the Rush) hypothesis^10^.

We found a fluctuating relationship between body mass and position of species on the slow–fast continuum axis and risk-taking at different stages of the annual cycle. Larger-bodied species take fewer risks during breeding (possibly as parental birds are over-exposed at this time) and (marginally) also during winter, but this relationship disappeared during migration. Risk-taking is usually found to be strongly negatively related to body mass (i.e. large-sized species have longer FID)^5,9^, a fact that has been attributed to several physiological, ecological and behavioural mechanisms^39,40^. Nevertheless, most studies have been conducted during the breeding season. The fitness value of slow-lived species depends on the length of a reproductive life while fitness of fast-lived animals is strongly linked to the investments to current reproduction^38^. We failed to find a relationship between slow–fast continuum (i.e. survival rate) and risk-taking except during breeding; this may be due to low power of the analysis associated with relatively low number of species, especially for migrating birds. However, survival rate appears to be season-dependent in some migrants and thus could be associated with a wide range of time- and space-specific processes^56^. Lack of overall pattern during the annual cycle emphasise that different selective pressures may produce stage-specific differential responses over the cycle. Altogether, while position of species on slow–fast continuum axis remains good predictor of escape strategy during breeding, its effect on risk-taking may be strongly mitigated mainly during the migration by the need to increase risk-taking in order to meet high food intake requirements.

## Conclusion

To the best of our knowledge, this is the first study providing empirical evidence for temporal variation in risk-taking in wild-living migratory animals during the main stages of their annual cycle. Risk-taking in waders and the relationship between risk-taking and life-history traits changed seasonally, probably reflecting an adjustment in antipredator strategy in response to the costs and benefits of escape in fluctuating environments. Therefore, waders may respond to variable environments in complex ways with behavioural plasticity presumably playing a crucial role when coping with these challenges. As such, our results may have important consequences for future studies of cost-benefit trade-offs between risk-taking and animal traits in fluctuating environment, and suggesting that behaviour can be considered as a part of life-history^41^. They may also provide a framework for understanding present season-linked variation in research on risk-related behaviour. It remains unclear whether our findings could be applicable also for other migration systems (e.g. savannah, high-latitude and altitudinal migratory species) or whether animals with different life-history traits exhibit different levels of temporal variation in risk-taking. Finally, understanding behavioural changes across seasons is essential to help conservation of (often threatened) long-distant migrants, e.g. to determine buffer zones around them^57^.

## Material and Methods

### Study sites

Fieldwork took place during the breeding (April–August 2006–2010, 2012–2016) and wintering periods (January and February 2015–2016) and at stopover sites during spring migration (February and March 2016). Data for breeding birds were mainly recorded in northern and north-western Europe, including sites in Finland, Denmark, Sweden, Norway and Germany. Few other records were obtained from Spain, France, Macedonia and Ukraine. Stopover FID data were collected in Eilat, Israel, which is considered to be a major stop-over site for many western, central and northern European populations of waders and other avian migrants, including many of the studied breeding populations and species^24,48,51,58–62^. Israel and Sinai are a major land-bridge joining Eurasian breeding grounds with African wintering grounds. Water quality and composition have been maintained constant throughout the year during the last two decades (R. Yosef unpubl. data), limiting the possibility that our data could be biased, for instance, by exceptionally poor food conditions during the year when data were collected. In addition, large variance in body masses of waders ringed in previous seasons indicates that individuals in both poor, intermediate and very good body condition use Eilat as a stopover site (cf.^24,62–64^). This suggests that it is not an emergency stopover site only being used when birds achieve very poor body condition, but a regular stopover site for all migrating birds, a result further supported by long-term ringing programs clearly showing that waders can stay for longer periods at the site^59,61^. Wintering birds were examined in coastal areas and inland waterbodies of Kenya and Uganda (for localities see Appendix 1).

### Flight initiation distance (FID)

The FID of an animal when approached by a human is a widely used estimate of the level of risk-taking^4,5,9,36^. The distance at which individual birds escape is a reliable measure of risk-taking and is considered to reflect the trade-off between benefits associated with staying put and the costs of disturbance (e.g. stop feeding, defending territory and/or energetic costs of flight). Large FID-values indicate low risk-taking, whereas small FID-values indicate great risk-taking.

FID data were collected using a standardised procedure^5,9,30^. Briefly, when an individual bird had been spotted by a researcher using a pair of binoculars, the researcher moved at a normal walking speed directly towards the bird while recording the number of ~1 m steps. The FID represents the distance between the observer and the bird when it first started to escape. We only focused on birds on the ground and in open spaces (at least 20 m from cover), thus avoiding problems linked with shrub and tree cover significantly decreasing FID^34^ and minimising problems maintaining a constant walking speed at localities in differing habitats. The study species of waders are in general open habitat species throughout their annual cycle^42^. To eliminate effects of habitat openness on FID, most birds were approached in open areas (study sites were mostly represented by sand and rocky coasts and shores of lakes/water bodies, river banks and open agricultural fields with no or sparse vegetation cover). Moreover, all FID data were collected during favourable weather conditions, i.e. on sunny days with no or only moderate wind. Furthermore, we only focused on birds that were foraging or engaged in comfort behaviour, such as roosting or preening; birds sitting on the nest or birds exhibiting highly vigilant behaviour were discarded from the dataset. When approaching a bird, researchers wore standardised outdoor clothing with no bright colours. FID data were taken by the following researchers in each region: Germany, Macedonia and Sweden (PM), Norway (APM, PM), Denmark, France, Spain and Ukraine (APM), Finland (JJ, M-LK-J), Israel (GK, RY), Kenya (PM, MH) and Uganda (PT). All researchers have a long-term experience to do flight-initiation distance measurements by using these standardized protocols.

Breeding populations tend to be mostly sedentary, with only small territories defended and birds roaming no more than a few hundred metres from the nest^65^. After each sampling event, therefore, we moved to another site (at least few hundred meters away) to avoid sampling the same individual twice. As the birds examined were not banded, we cannot completely exclude the possibility that some individuals on the breeding grounds may have been late migrants. When sampling birds on migration, we approached birds at the same locality several times (but with >24-hour interval between sampling events) as individual turn-over at such sites is high^52,53^. The same method of data collection was used for wintering birds as even the smallest wintering home ranges greatly exceed that of breeding birds^66,67^. Sampling was designed to minimize repeated sampling of the same individuals. Nevertheless, it has been found that even a modest degree of re-sampling of the same individuals does not influence the results^68^. To ensure that species-specific FID was not influenced by the response of other bird species, we approached single-species groups only. In flock cases, we selected the closest individual of the flock to the observer when estimating the FID.

We also estimated ‘starting distance’ (hereafter SD) as the distance between the initial location of a bird and the location where we first spotted it and started walking towards the individual. SD is often correlated with FID^9,36,69^, resulting in problems with collinearity. We partly eliminated this problem by performing the majority of measurements by approaching birds >30 m away from the observer^5,36^, and by statistically controlling for SD effects by including it as a covariate in all analyses.

### Covariates

#### Flock size

Flock size was estimated as the number of individuals feeding or roosting together in a monospecific flock, separated (by > 20 metres) from single birds or other flocks. Flock-size data at breeding sites were collected before individuals dispersed from their breeding territories.

#### Body mass

As species-specific FID has been shown to be strongly positively related to body mass^5,9^, we obtained information on body mass (in g) from the online edition of ‘Handbook of the Birds of the World’^70^. When sex-specific mass was provided, we used the average value for the species.

#### Clutch mass

Data on clutch size were obtained from the supplementary material in Jetz *et al*.^71^. Information on fresh egg mass was primarily obtained from the supplementary material in Liker *et al*.^72^, with missing species data added from other sources^73,74^. Clutch mass was then calculated as clutch size x egg mass.

#### Survival rate

Most data on adult survival rate were primarily obtained from Cramp & Simmons^43^, with missing species data added from other sources^75–78^. If multiple estimates were available (e.g. for several populations), we calculated the weighted mean based on sample size or the midpoint when only ranges were presented. For some species, however, survival rate was not available. To increase power of analysis, we assigned to *Tringa erythropus* missing information on survival, the survival value of the species most similar in ecology and size, *T*. *totanus*.

#### Human activity

Because the level of human presence may affect risk-taking behaviour of animals directly or indirectly^34,57^, each site was scored with respect to the number of different human individuals counted during a session: 0 = low (1–4 per session), 1 = moderate (5–10 per session) and 2 = high human activity (>11 per session).

### Statistical analysis

Only species recorded in at least two stages of their annual migration cycle (e.g. breeding and migration) were included in statistical analysis. Prior to analysis, FID, SD and flock size were log-transformed to meet normality and homoscedasticity requirements following Zar^79^, with mean values for populations used as sampling units. In all analyses, we assumed that estimates based on larger sample sizes were closer to the true population estimate and, therefore, all analyses were weighted by sample size to adjust for unequal sampling effort between species^80^.

Effects of common phylogenetic descent may bias results such that different observations will not be statistically independent due to shared phylogenetic history. Pagel’s λ varies between 0 and 1; the phylogenetic scaling parameter λ of 0 indicates that a trait is random with respect to phylogeny (i.e. phylogenetic independence), whereas a λ of 1 indicates phylogenetic trait conservatism (i.e. phylogenetic dependence)^81^. In order to test whether *λ* differed from 0, we used phylogenetic generalized least square regression (PGLS) models as implemented in the R statistical environment, using the *ape*, *MASS* and *mvtnorm* packages and the *pglm3.3.r* function (Appendix 2). Different populations of the same species were treated as polytomies with a constant small genetic distance of 1∙10^−5^ between conspecific populations (see Díaz *et al*.^5^ for a similar approach). We obtained the consensus tree for bird species with available data (Appendix 3) using Mesquite software^82^ on 100 trees extracted from the phylogeny published by Jetz *et al*.^83^. The inverse of sample sizes was used to correct for sampling effort^84^.

We first tested for potential effects of confounding variables (SD, flock size and human activity) on FID. Then, we tested for effects of covariates on differences in FID between stages of the annual cycle after controlling for confounding variables. Finally, we tested for life history effects on FID, and whether these effects varied among stages of the life cycle, by including life history x stage interactions in the PGLS models. Body and clutch mass and survival rate are strongly correlated due to both physiological design factors and to shared phylogenetic history^41^, so that including them as independent covariates may give erroneous results. Life history traits were thus combined by means of phylogenetic principal component analyses (PPCA)^85^ to account for their pattern of correlation. We built two phylogenetically corrected principal components (PCs) explaining 66% of the original variance in the data set (λ = 0.942, χ^2^_19_ = 4.94. P < 0.001). PC1 was negatively associated with body mass and clutch mass (r = −0.95 and r = −0.96, respectively; % variance = 33%) and can thus be interpreted as an inverse gradient of body mass independent to survival rate. PC2 can be interpreted as a slow–fast pace of life gradient^41^ inversely associated with adult survival (r = −0.88) and only weakly correlated with body and clutch mass (r = 0.25 and r = 0.19, respectively; % variance = 33%). Factor scores (corrected for phylogeny) of each bird species are shown in Appendix 1. Effect sizes were computed as r statistics from P values of t-tests^86^. Effect sizes were judged as small (Pearson r < 0.10, explaining <1% of the variance), intermediate (r = 0.11–0.49, 9–24% of the variance) and large (r > 0.50, 25% or more of the variance)^87^.

### Ethical statement

No permits are required for this kind of research in Europe and Israel. Tropical research was approved by National Commission for Science Technology and Innovation in Kenya (NACOSTI/P/14/4653/660). All methods used in the study were carried out in accordance with the approved guidelines. FID data were only collected in public spaces and private land where no special permit was required. This method is designed to cause only brief and minimal disturbance to birds, and this method does not differ from standard background disturbance caused by other visitors.

## Electronic supplementary material

Supplementary material

## Data Availability

The datasets supporting this article have been uploaded as part of the supplementary material.
