# Supplementary material for: Adjusting risk-taking to the annual cycle of long-distance migratory birds
Source: Sci Rep. 2018 Sep 18;8:13989. doi: 10.1038/s41598-018-32252-1 (PMC6143617; doi:10.1038/s41598-018-32252-1)
Supplement: Supplementary file 1 — Supplementary material [file 41598_2018_32252_MOESM1_ESM.pdf]

Supplementary material

Adjusting risk taking to the annual cycle of long-distance migratory birds

Peter Mikula, Mario Díaz, Tomáš Albrecht, Jukka Jokimäki, Marja-Liisa Kaisanlahti-Jokimäki, Gal Kroitero, Anders Pape Møller, Piotr Tryjanowski, Reuven Yosef, Martin Hromada

Supplementary Table S1. Results of a phylogenetic generalised least square regression (PGLS) model testing for the confounding effects of starting distance, flock size and human activity on flight initiation distance (FID) after accounting for the phylogenetic ($\lambda = 0.415$; $\chi^2_{112} = 8.29$, P = 0.004) and sample size (V+5000W) effects. Model statistics are $F_{4, 112} = 40.17$, $P < 0.001$, $R^2 = 0.51$, AIC = -87.8. Boldface indicates statistical significance, and Pearson r values effect sizes.

**Effect**	**B**	**SE(B)**	**t**	**P**	**r**
Starting distance	0.50	0.05	10.39	**<0.001**	0.609
Flock size	-0.11	0.03	-3.91	**<0.001**	0.349
Human activity	-0.02	0.02	-1.31	0.1933	0.124

Appendix 1. Summary statistics. Information on bird species and populations, stage of cycle, study sites, human activity on localities, flock size, starting distance (m), mean flight initiation distance (FID) (m), standard deviation FID (m), minimum and maximum FID, sample size (N), body mass (g), clutch size (No.), egg mass (g) and survival rate (%) and PC1 and PC2 scores for the 115 sampled populations.

Species	Stage	Country	Locality	Human activity	Flock size	Starting distance	FID	Standard deviation FID	Min	Max	N	Body size	Clutch size	Egg mass	Survival rate	PC1	PC2
Actitis_hypoleucos	Breeding	Denmark	Brønderslev	0	1	30.4	22.2	5.83	12	33	12	63.0	4	12.5	75.0	6.17	-5.45
Actitis_hypoleucos	Breeding	Finland	Rovaniemi	0	1	21.6	19.8	12.58	7	40	5	63.0	4	12.5	75.0	6.17	-5.45
Actitis_hypoleucos	Breeding	France	Paris	0	1	30.0	20.0	0.00	20	20	1	63.0	4	12.5	75.0	6.17	-5.45
Actitis_hypoleucos	Breeding	Norway	Dovrefjell	0	1	52.0	34.3	3.51	31	38	3	63.0	4	12.5	75.0	6.17	-5.45
Actitis_hypoleucos	Breeding	Spain	Barcelona	0	1	30.0	16.7	9.87	10	28	3	63.0	4	12.5	75.0	6.17	-5.45
Actitis_hypoleucos	Breeding	Sweden	Ystad	1	2	41.3	19.8	6.40	13	27	4	63.0	4	12.5	75.0	6.17	-5.45
Arenaria_interpres	Breeding	Denmark	Brønderslev	0	1	40.0	20.5	6.36	16	25	2	137.0	4	17.9	77.8	2.48	-5.47
Arenaria_interpres	Breeding	Sweden	Ystad	1	2	58.0	29.0	0.00	29	29	1	137.0	4	17.9	77.8	2.48	-5.47
Burhinus_oedicnemus	Breeding	Denmark	Brønderslev	0	1	60.0	45.0	0.00	45	45	1	412.5	2	42.0	83.2	-6.09	-4.72
Calidris_alba	Breeding	Denmark	Brønderslev	0	1	30.0	16.9	3.35	10	23	55	75.0	4	11.2	56.0	8.96	2.56
Calidris_alpina	Breeding	Denmark	Brønderslev	0	1	50.0	22.0	0.00	22	22	1	59.5	4	10.7	75.0	6.73	-5.62
Calidris_alpina	Breeding	Sweden	Ystad	1	6	53.0	20.3	1.15	19	21	3	59.5	4	10.7	75.0	6.73	-5.62
Calidris_minuta	Breeding	Denmark	Brønderslev	0	1	30.0	19.6	5.24	12	30	42	59.5	4	10.7	75.0	6.73	-5.62
Himantopus_himantopus	Breeding	Spain	Barcelona	0	1	30.0	22.5	0.58	22	23	4	185.5	4	21.8	70.0	1.36	-1.41
Charadrius_alexandrinus	Breeding	Germany	Heligoland	2	1	31.0	10.0	0.00	10	10	1	44.0	3	9.0	60.0	10.33	0.23
Charadrius_alexandrinus	Breeding	Spain	Barcelona	0	1	30.0	9.5	1.52	8	12	6	44.0	3	9.0	60.0	10.33	0.23
Charadrius_dubius	Breeding	Finland	Rovaniemi	0	1	31.0	18.0	0.00	18	18	1	39.5	4	7.7	45.0	12.37	6.57
Charadrius_dubius	Breeding	Spain	Barcelona	0	1	30.0	9.0	0.00	9	9	1	39.5	4	7.7	45.0	12.37	6.57
Charadrius_dubius	Breeding	Sweden	Öland	2	1	44.6	18.1	3.44	13	24	7	39.5	4	7.7	45.0	12.37	6.57
Charadrius_dubius	Breeding	Ukraine	Pripyat	0	1	30.0	20.0	2.83	18	22	2	39.5	4	7.7	45.0	12.37	6.57
Charadrius_hiaticula	Breeding	Denmark	Brønderslev	0	1	31.6	21.1	7.02	10	40	37	60.0	3.5	11.5	58.0	9.35	1.49
Charadrius_hiaticula	Breeding	Finland	Rovaniemi	0	1	36.6	20.4	8.17	10	30	5	60.0	3.5	11.5	58.0	9.35	1.49
Charadrius_hiaticula	Breeding	Germany	Heligoland	2	1	66.0	19.5	3.54	17	22	2	60.0	3.5	11.5	58.0	9.35	1.49

Charadrius_hiaticula	Breeding	Norway	Runde	1	1	53.2	29.2	6.24	19	37	6	60.0	3.5	11.5	58.0	9.35	1.49
Charadrius_hiaticula	Breeding	Sweden	Ystad	1	2	45.0	9.0	0.00	9	9	1	60.0	3.5	11.5	58.0	9.35	1.49
Limosa_limosa	Breeding	Denmark	Brønderslev	0	1	50.0	47.0	0.00	47	47	1	330.0	4	39.0	63.1	-5.80	4.26
Numenius_arquata	Breeding	Denmark	Brønderslev	0	1	67.0	57.6	13.75	40	70	10	710.0	4	76.0	73.6	-26.63	6.48
Numenius_arquata	Breeding	Finland	Rovaniemi	0	1	57.6	36.9	14.05	19	60	11	710.0	4	76.0	73.6	-26.63	6.48
Numenius_arquata	Breeding	Germany	Otterndorf	0	5	227.0	44.0	8.66	39	54	3	710.0	4	76.0	73.6	-26.63	6.48
Numenius_phaeopus	Breeding	Denmark	Brønderslev	0	1	50.0	38.0	0.00	38	38	1	434.0	4	50.0	69.2	-12.20	3.58
Numenius_phaeopus	Breeding	Finland	Rovaniemi	0	2	188.0	30.0	14.14	20	40	2	434.0	4	50.0	69.2	-12.20	3.58
Philomachus_pugnax	Breeding	Denmark	Brønderslev	0	1	50.0	20.0	0.00	20	20	1	162.0	4	21.0	52.4	4.71	5.70
Pluvialis_squatarola	Breeding	Denmark	Brønderslev	1	1	80.0	51.0	21.21	36	66	2	280.0	4	34.2	76.0	-5.15	-2.04
Tringa_erythropus	Breeding	Denmark	Brønderslev	0	1	30.0	23.0	4.36	20	28	3	163.5	4	24.5	71.3	1.02	-1.97
Tringa_erythropus	Breeding	Macedonia	Prespa	0	1	62.0	31.0	0.00	31	31	1	163.5	4	24.5	71.3	1.02	-1.97
Tringa_glareola	Breeding	Denmark	Brønderslev	0	1	30.0	20.3	3.79	16	23	3	66.0	4	13.5	53.6	8.93	3.66
Tringa_glareola	Breeding	Sweden	Skuleskogen	1	1	51.0	23.5	6.36	19	28	2	66.0	4	13.5	53.6	8.93	3.66
Tringa_nebularia	Breeding	Denmark	Brønderslev	1	1	41.5	37.8	13.57	20	53	4	212.5	4	30.5	82.0	-3.34	-5.52
Tringa_nebularia	Breeding	Finland	Rovaniemi	0	1	55.0	46.5	53.03	9	84	2	212.5	4	30.5	82.0	-3.34	-5.52
Tringa_nebularia	Breeding	Norway	Oslo	0	1	30.0	30.0	0.00	30	30	1	212.5	4	30.5	82.0	-3.34	-5.52
Tringa_nebularia	Breeding	Norway	Runde	1	2	40.0	22.0	0.00	22	22	1	212.5	4	30.5	82.0	-3.34	-5.52
Tringa_ochropus	Breeding	Denmark	Brønderslev	0	1	65.0	38.0	9.90	31	45	2	86.0	4	30.5	84.0	-0.42	-7.64
Tringa_ochropus	Breeding	Finland	Rovaniemi	0	1	12.0	10.0	0.00	10	10	1	86.0	4	30.5	84.0	-0.42	-7.64
Tringa_ochropus	Breeding	Norway	Bømlo	1	2	29.0	16.0	0.00	16	16	1	86.0	4	30.5	84.0	-0.42	-7.64
Tringa_ochropus	Breeding	Norway	Stord	0	3	85.0	45.7	8.50	37	54	3	86.0	4	30.5	84.0	-0.42	-7.64
Tringa_totanus	Breeding	Denmark	Brønderslev	0	1	39.6	26.5	10.29	12	40	13	120.0	4	22.3	71.3	2.70	-2.58
Tringa_totanus	Breeding	Germany	Otterndorf	0	1	68.5	31.5	4.95	28	35	2	120.0	4	22.3	71.3	2.70	-2.58
Tringa_totanus	Breeding	Norway	Runde	1	1	107.1	40.3	5.15	33	48	7	120.0	4	22.3	71.3	2.70	-2.58
Tringa_totanus	Breeding	Spain	Barcelona	0	1	30.0	18.0	0.00	18	18	1	120.0	4	22.3	71.3	-0.42	-7.64
Tringa_totanus	Breeding	Sweden	Ystad	1	2	47.0	23.3	2.52	21	26	3	120.0	4	22.3	71.3	2.70	-2.58
Calidris_alpina	Migration	Israel	Eilat	0	3	49.1	19.8	7.14	4	30	56	59.5	4	10.7	75.0	6.73	-5.62
Calidris_minuta	Migration	Israel	Eilat	0	10	28.1	13.8	6.06	4	39	512	59.5	4	10.7	75.0	6.73	-5.62

Himantopus_himantopus	Migration	Israel	Eilat	0	5	34.4	15.7	9.05	2	70	519	185.5	4	21.8	70.0	1.36	-1.41
Charadrius_alexandrinus	Migration	Israel	Eilat	0	2	13.5	8.3	2.94	6	12	6	44.0	3	9.0	60.0	10.33	0.23
Charadrius_dubius	Migration	Israel	Eilat	0	6	24.1	8.7	5.98	3	25	10	39.5	4	7.7	45.0	10.33	0.23
Charadrius_hiaticula	Migration	Israel	Eilat	0	18	35.0	14.5	4.51	8	18	4	60.0	3.5	11.5	58.0	9.35	1.49
Numenius_arquata	Migration	Israel	Eilat	0	2	27.6	13.1	6.96	5	22	7	710.0	4	76.0	73.6	-26.63	6.48
Tringa_erythropus	Migration	Israel	Eilat	0	2	25.2	13.2	3.43	8	17	6	163.5	4	24.5	71.3	1.02	-1.97
Tringa_glareola	Migration	Israel	Eilat	0	5	39.5	18.3	11.21	4	40	13	66.0	4	13.5	53.6	8.93	3.66
Tringa_totanus	Migration	Israel	Eilat	0	3	33.7	18.0	9.54	2	40	310	120.0	4	22.3	71.3	2.70	-2.58
Actitis_hypoleucos	Wintering	Kenya	El Molo	2	1	67.7	15.2	3.05	11	21	10	63.0	4	12.5	75.0	6.17	-5.45
Actitis_hypoleucos	Wintering	Kenya	Koobi Fora	0	1	61.7	18.3	3.52	14	26	15	63.0	4	12.5	75.0	6.17	-5.45
Actitis_hypoleucos	Wintering	Kenya	Loiyangalani	0	2	55.9	13.4	4.79	5	22	19	63.0	4	12.5	75.0	6.17	-5.45
Actitis_hypoleucos	Wintering	Kenya	Naivasha	1	1	49.2	18.0	5.43	11	24	5	63.0	4	12.5	75.0	6.17	-5.45
Actitis_hypoleucos	Wintering	Kenya	Thomson's Falls	2	1	33.0	12.0	0.00	12	12	1	63.0	4	12.5	75.0	6.17	-5.45
Actitis_hypoleucos	Wintering	Kenya	Watamu	2	3	63.3	15.3	6.65	6	21	4	63.0	4	12.5	75.0	6.17	-5.45
Actitis_hypoleucos	Wintering	Uganda	Mburo	0	1	41.0	12.0	0.00	12	12	1	63.0	4	12.5	75.0	6.17	-5.45
Arenaria_interpres	Wintering	Kenya	Mida Creek	0	2	183.0	39.0	0.00	39	39	1	137.0	4	17.9	77.8	2.48	-5.47
Burhinus_oedicnemus	Wintering	Kenya	Koobi Fora	0	2	51.0	22.0	0.00	22	22	1	412.5	2	42.0	83.2	-6.09	-4.72
Calidris_alba	Wintering	Kenya	Loiyangalani	0	2	43.5	14.0	1.41	13	15	2	75.0	4	11.2	56.0	8.96	2.56
Calidris_alba	Wintering	Kenya	Watamu	2	4	102.5	26.5	4.95	23	30	2	75.0	4	11.2	56.0	8.96	2.56
Calidris_minuta	Wintering	Kenya	El Molo	2	38	72.6	23.0	7.16	14	41	11	59.5	4	10.7	75.0	6.73	-5.62
Calidris_minuta	Wintering	Kenya	Koobi Fora	0	2	66.2	18.2	4.63	11	34	29	59.5	4	10.7	75.0	6.73	-5.62
Calidris_minuta	Wintering	Kenya	Loiyangalani	0	9	65.7	15.0	2.65	12	17	3	59.5	4	10.7	75.0	6.73	-5.62
Calidris_minuta	Wintering	Kenya	Mida Creek	0	37	147.5	43.0	6.63	32	53	8	59.5	4	10.7	75.0	6.73	-5.62
Calidris_minuta	Wintering	Kenya	Naivasha	1	1	61.3	28.0	5.00	23	33	3	59.5	4	10.7	75.0	6.73	-5.62
Calidris_minuta	Wintering	Kenya	Olbolosat	1	1	55.9	27.7	7.45	18	44	9	59.5	4	10.7	75.0	6.73	-5.62
Calidris_minuta	Wintering	Kenya	Watamu	2	12	137.0	25.5	12.02	17	34	2	59.5	4	10.7	75.0	6.73	-5.62
Himantopus_himantopus	Wintering	Kenya	El Molo	2	1	99.0	26.3	2.31	25	29	3	185.5	4	21.8	70.0	1.36	-1.41
Himantopus_himantopus	Wintering	Kenya	Koobi Fora	0	3	79.7	32.7	9.64	20	53	15	185.5	4	21.8	70.0	1.36	-1.41
Himantopus_himantopus	Wintering	Kenya	Naivasha	1	1	66.0	27.0	3.00	24	30	3	185.5	4	21.8	70.0	1.36	-1.41

Charadrius_alexandrinus	Wintering	Kenya	Mida Creek	0	1	166.0	22.0	0.00	22	22	1	44.0	3	9.0	60.0	10.33	0.23
Charadrius_dubius	Wintering	Kenya	El Molo	2	1	32.0	12.0	0.00	12	12	1	39.5	4	7.7	45.0	12.37	6.57
Charadrius_dubius	Wintering	Kenya	Loiyangalani	0	1	77.5	12.0	1.41	11	13	2	39.5	4	7.7	45.0	12.37	6.57
Charadrius_dubius	Wintering	Kenya	Watamu	2	1	65.8	17.8	2.86	14	22	6	39.5	4	7.7	45.0	12.37	6.57
Charadrius_hiaticula	Wintering	Kenya	El Molo	2	2	45.4	15.9	4.20	10	25	10	60.0	3.5	11.5	58.0	9.35	1.49
Charadrius_hiaticula	Wintering	Kenya	Koobi Fora	0	2	55.8	16.9	3.92	11	24	27	60.0	3.5	11.5	58.0	9.35	1.49
Charadrius_hiaticula	Wintering	Kenya	Loiyangalani	0	4	60.1	15.7	3.63	9	22	19	60.0	3.5	11.5	58.0	9.35	1.49
Charadrius_hiaticula	Wintering	Kenya	Mida Creek	0	21	107.7	24.0	10.30	13	40	6	60.0	3.5	11.5	58.0	9.35	1.49
Charadrius_hiaticula	Wintering	Kenya	Naivasha	1	1	59.8	30.5	4.43	26	36	4	60.0	3.5	11.5	58.0	9.35	1.49
Charadrius_hiaticula	Wintering	Kenya	Olbolosat	1	1	69.7	25.3	1.53	24	27	3	60.0	3.5	11.5	58.0	9.35	1.49
Charadrius_hiaticula	Wintering	Kenya	Watamu	2	4	74.1	16.1	6.31	10	29	14	60.0	3.5	11.5	58.0	9.35	1.49
Limosa_limosa	Wintering	Kenya	Koobi Fora	0	4	135.0	34.0	0.00	34	34	1	330.0	4	39.0	63.1	-5.80	4.26
Numenius_arquata	Wintering	Kenya	Mida Creek	0	1	251.0	50.0	5.66	46	54	2	710.0	4	76.0	73.6	-26.63	6.48
Numenius_phaeopus	Wintering	Kenya	Watamu	2	4	109.0	40.5	37.48	14	67	2	434.0	4	50.0	69.2	-26.63	6.48
Philomachus_pugnax	Wintering	Kenya	Koobi Fora	0	1	70.0	35.0	0.00	35	35	1	162.0	4	21.0	52.4	4.71	5.70
Philomachus_pugnax	Wintering	Kenya	Mida Creek	0	2	135.0	44.0	0.00	44	44	1	162.0	4	21.0	52.4	4.71	5.70
Philomachus_pugnax	Wintering	Kenya	Naivasha	1	1	64.1	26.6	5.16	20	36	7	162.0	4	21.0	52.4	4.71	5.70
Philomachus_pugnax	Wintering	Kenya	Olbolosat	1	1	70.0	33.4	6.73	24	41	9	162.0	4	21.0	52.4	4.71	5.70
Pluvialis_squatarola	Wintering	Kenya	Mida Creek	0	4	143.3	41.1	7.61	32	53	8	280.0	4	34.2	76.0	-5.15	-2.04
Pluvialis_squatarola	Wintering	Kenya	Watamu	2	4	159.0	37.0	0.00	37	37	1	280.0	4	34.2	76.0	-5.15	-2.04
Tringa_glareola	Wintering	Kenya	Koobi Fora	0	1	61.3	24.7	6.66	19	32	3	66.0	4	13.5	53.6	8.93	3.66
Tringa_glareola	Wintering	Kenya	Mida Creek	0	5	236.0	37.0	6.98	31	46	4	66.0	4	13.5	53.6	8.93	3.66
Tringa_glareola	Wintering	Kenya	Olbolosat	1	2	69.3	26.0	2.00	24	28	3	66.0	4	13.5	53.6	8.93	3.66
Tringa_glareola	Wintering	Uganda	Mburo	0	1	39.0	20.5	3.54	18	23	2	66.0	4	13.5	53.6	8.93	3.66
Tringa_nebularia	Wintering	Kenya	El Molo	2	1	95.1	34.0	9.90	21	45	7	212.5	4	30.5	82.0	-3.34	-5.52
Tringa_nebularia	Wintering	Kenya	Koobi Fora	0	1	175.6	23.4	5.27	15	29	10	212.5	4	30.5	82.0	-3.34	-5.52
Tringa_nebularia	Wintering	Kenya	Loiyangalani	0	1	71.0	21.0	4.98	16	27	6	212.5	4	30.5	82.0	-3.34	-5.52
Tringa_nebularia	Wintering	Kenya	Mida Creek	0	11	135.2	38.2	5.31	33	51	14	212.5	4	30.5	82.0	-3.34	-5.52
Tringa_nebularia	Wintering	Kenya	Watamu	2	2	80.8	29.4	7.11	18	37	9	212.5	4	30.5	82.0	-3.34	-5.52

Tringa_ochropus	Wintering	Kenya	Loiyangalani	0	2	61.0	17.0	2.83	15	19	2	86.0	4	30.5	84.0	-0.42	-7.64
Tringa_ochropus	Wintering	Kenya	Mida Creek	0	5	249.0	36.5	6.86	31	46	4	86.0	4	30.5	84.0	-0.42	-7.64
Tringa_ochropus	Wintering	Uganda	Tororo	0	1	45.0	8.0	0.00	8	8	1	86.0	4	30.5	84.0	-0.42	-7.64
Tringa_totanus	Wintering	Kenya	Mida Creek	0	3	128.0	38.7	2.52	36	41	3	120.0	4	22.3	71.3	2.70	-2.58
Tringa_totanus	Wintering	Kenya	Naivasha	1	2	60.0	24.0	2.83	22	26	2	120.0	4	22.3	71.3	2.70	-2.58

Appendix 2. R scripts used to test for differences in mean flight initiation distance (FID) among stages of the annual cycle (breeding, migration, and wintering grounds) after controlling for phylogeny (lambda) and sample sizes ($1/N_i$ values arranged in a Wvector W). Model 1 tested for the effects of the confounding covariates human activity (HUMAN), starting distance (LOGSD) and flock size (LOGFLOCK) on FID. Model 2 tested for differences in FID among stages (STAGE) after accounting for effects of confounding covariates, and model 3 for effects of the life-history traits body mass, clutch mass (clutch size x egg mass) and survival rate after its combination into two Principal Components (PC1 and PC2) by means of phylogenetic principal component analyses. Model 3 tested for both pure effects of covariates and for their variation among stages of the annual cycle (STAGE*covariate terms).

```
library(MASS)
library(mvtnorm)
library(ape)
source("C:/R/pglm3.3.r")
setwd("C:/R/")
fa<-read.tree("PHYLOGENY.txt")
V<-vcv.phylo(fa)
data<-read.table("DATA",header=T)
attach(data)
fix(data)

mod1<-pglmEstLambda(LOGFID~HUMAN+LOGSD+LOGFLOCK,data=data, V)
summary(mod1)

mod2<-pglmEstLambda(LOGFID~LOGSD+LOGFLOCK+STAGE,data=data, V)
summary(mod2)

mod3<-
pglmEstLambda(LOGFID~LOGSD+LOGFLOCK+PC1+PC2+STAGE+PC1*STAGE+
PC2*STAGE,data=data, V)
summary(mod3
```

Wvector<-c(1/N_1,

1/N_2,

......,

1/N_n)

W <- diag(Wvector)

rownames(W) <- rownames(V)

mod10<-pglm(LOGFID~LOGSD+LOGFLOCK+PC1+PC2+STAGE+PC1*STAGE+PC2*STAGE, data = data, V + 1*W, lambda = Lambda)

summary(mod10)

References for R packages:

Genz A., Bretz F., Miwa T., Mi X., Leisch F., Scheipl F. & Hothorn T. mvtnorm: Multivariate Normal and t Distributions. R package version 1.0-6. URL: http://CRAN.R-project.org/package=mvtnorm (2017).

Paradis E., Claude J. & Strimmer K. APE: analyses of phylogenetics and evolution in R language. *Bioinformatics* **20**, 289–290 (2004).

Revell L. J. Size-correction and principal components for interspecific comparative studies. *Evolution* **63**, 3258–3268 (2009).

Venables W. N. & Ripley B. D. *Modern Applied Statistics with S. Fourth Edition*. (Springer, 2002).

Appendix 3. Phylogenetic relationships among the analysed species of waders.

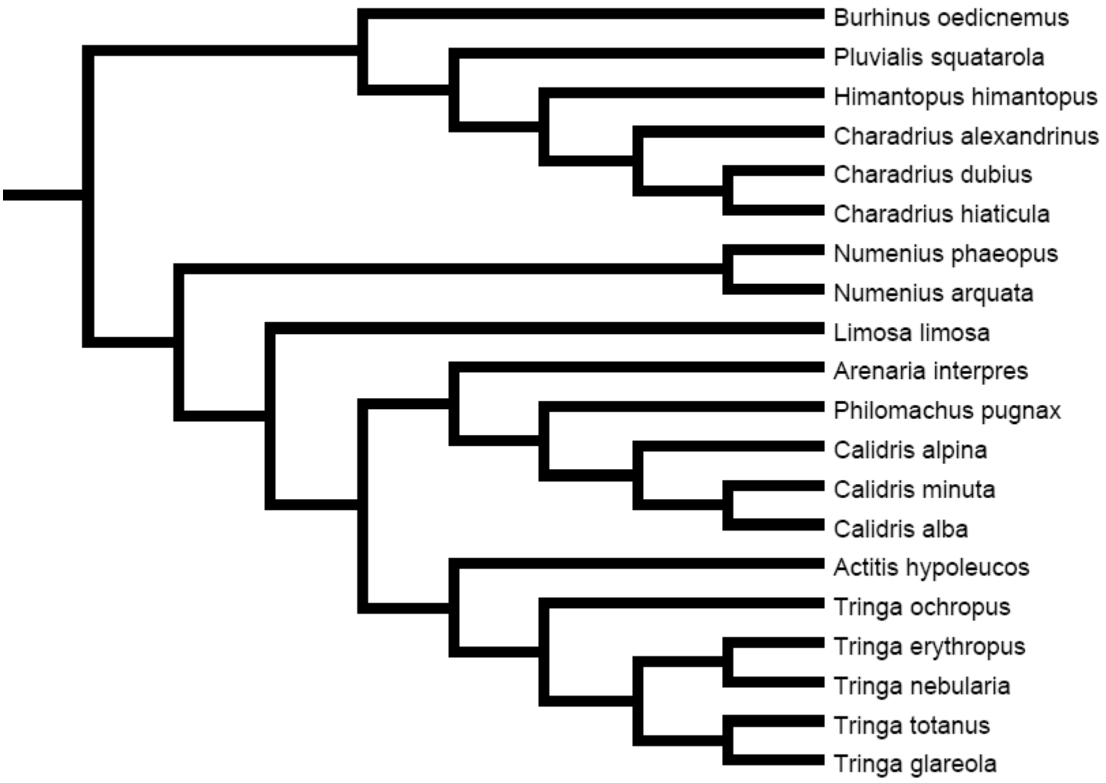

((Burhinus_oedicnemus:57.69839803,(Pluvialis_squatarola:45.9961262,(Himantopus_h
imantopus:43.135072480000005,(Charadrius_alexandrinus:35.75475004000002,(Chara
drius_dubius:23.895144910000013,Charadrius_hiaticula:23.895144970000004):5.9298
026849999985):3.6901609600000014):1.4305271):5.85113588):5.299793220000001,((
Numenius_phaeopus:14.527597580000002,Numenius_arquata:14.527597460000003):1
2.266474699999998,(Limosa_limosa:35.06059179,((Arenaria_interpres:26.755496679
999993,(Philomachus_pugnax:17.557721979999993,(Calidris_alpina:13.23101037,(Cal
idris_minuta:7.1249112100000005,Calidris_alba:7.12021641):6.17251908080808):4.32
201692):4.598887324999998):5.684327790000001,(Actitis_hypoleucos:27.264562990
000005,(Tringa_ochropus:19.45237948000001,((Tringa_erythropus:10.8910196399999
98,Tringa_nebularia:10.8910197):1.6884457549999996,(Tringa_totanus:7.6447408600
00002,Tringa_glareola:7.644740810000003):6.623170419999999):2.592234175):7.812
183500000002):2.587630715000001):1.3103837300000007):3.9999550500000014):23
.93764437);
